# Microfluidics Integration into Low-Noise Multi-Electrode Arrays

**DOI:** 10.3390/mi12060727

**Published:** 2021-06-20

**Authors:** Mafalda Ribeiro, Pamela Ali, Benjamin Metcalfe, Despina Moschou, Paulo R. F. Rocha

**Affiliations:** 1 Centre for Accountable, Responsible, and Transparent AI (ART-AI), Department of Computer Science, University of Bath, Bath BA2 7AY, UK; mr611@bath.ac.uk; 2 Centre for Biosensors, Bioelectronics, and Biodevices (C3Bio), Department of Electronic and Electrical Engineering, University of Bath, Bath BA2 7AY, UK; pamela7ali@gmail.com (P.A.); B.W.Metcalfe@bath.ac.uk (B.M.); 3 Centre for Functional Ecology (CFE), Department of Life Sciences, University of Coimbra, 3000-456 Coimbra, Portugal

**Keywords:** MEA, brain cells, electrical recordings, Organ-on-Chip, microfluidics, Brain-on-Chip

## Abstract

Organ-on-Chip technology is commonly used as a tool to replace animal testing in drug development. Cells or tissues are cultured on a microchip to replicate organ-level functions, where measurements of the electrical activity can be taken to understand how the cell populations react to different drugs. Microfluidic structures are integrated in these devices to replicate more closely an in vivo microenvironment. Research has provided proof of principle that more accurate replications of the microenvironment result in better micro-physiological behaviour, which in turn results in a higher predictive power. This work shows a transition from a no-flow (static) multi-electrode array (MEA) to a continuous-flow (dynamic) MEA, assuring a continuous and homogeneous transfer of an electrolyte solution across the measurement chamber. The process through which the microfluidic system was designed, simulated, and fabricated is described, and electrical characterisation of the whole structure under static solution and a continuous flow rate of 80 µL/min was performed. The latter reveals minimal background disturbance, with a background noise below 30 µVpp for all flow rates and areas. This microfluidic MEA, therefore, opens new avenues for more accurate and long-term recordings in Organ-on-Chip systems.

## 1. Introduction

The high cost of drug development commonly arises from the low success rate of clinically applicable drugs during the development stage. It takes on average 10–12 years [1] to develop a new drug, with two-thirds of the total costs ascribed to the clinical trial stage [2]. In order to reduce these costs and, thus, allow the more rapid introduction of effective drugs in clinical practice, it is critical that the accuracy and throughput of pre-clinical screening is significantly improved [3]. The traditional drug development route mostly relies on animal testing, which is costly, ethically contentious, and often offers poor predictive power for human response to drugs due to interspecific discrepancies [4]. 

Currently, the predictive power of alternative tissue models is compromised due to oversimplified cell microenvironments and tissue structures [5]. Micro-physiological systems, also known as Organ-on-Chip (OOC) technology, integrate microscale organ models on microchips, connected with microfluidic channels [6], delivering nutrients and reagents on-chip precisely and continuously, replicating tissue interactions within the body [7]. The in vitro cell culturing and microfluidics that are employed in this technology can emulate drug absorption, distribution, and metabolism in the human body much more accurately, increasing the potential of successful drug development. According to Wang et al. [5], the five core aspects of developing accurate OOC systems are a combination of the human physiology modelling and the microenvironment in which the cells are being cultured. These five areas are the controlled culture environment, pumpless microfluidic platforms, functional measurements, advanced single-organ models, and system integration.

Owing to its game-changing potential, OOC technology is currently being explored for various organ models. Brain-on-Chip technology, in particular, attempts to model and measure high-level brain functions which emerge from the interaction of interconnected neural networks [8]. Haring et al. highlighted the limitations of reductionist approaches and how they often fail to replicate the complexity and higher-order features of neural networks and the human nervous system. 

Measurements from the neural system are based on the electrical signalling associated with fluctuations of the membrane potential in individual cells [9]. These oscillations are a result of the bidirectional flow of Na^+^ and K^+^ ions in neurons, resulting in membrane potentials which reach tens of mVs when recorded intracellularly or hundreds of µVs when recorded extracellularly. Multi-electrode arrays (MEA) are used to measure the extracellular local field potentials of the cells that are adhered to microchip electrodes [10]. Using MEAs to measure extracellular bioelectrical activity in vitro enables the study of neuronal network processes, the electrophysiological mechanisms related to pathological diseases, and the effects of drugs on the cell populations [11]. Nonetheless, although neuronal firing is routinely recorded, extremely low-magnitude electrical signals, below 50 µV, still pose significant challenges in MEA recordings. 

An ultra-sensitive MEA capable of detecting the electrical activity of electrically quiescent cells such as rat C6 Glioma cells [9], breast cancer [12], and prostate cancer [13] has been previously developed. The microchip was made of a glass substrate with gold (Au) circular electrodes with a polymethyl methacrylate (PMMA) well glued on top of a substrate, serving as a container for the electrolyte solution. In addition to glass-based substrates, printed circuit board (PCB) approaches have also been implemented in the past to improve cost effectiveness, scalability to mass production, and future electronics integration of electrochemical devices [14]. Despite these advantages, it is important to note that PCBs traditionally use copper (Cu) for creating conductive traces and electrodes. A surface coating of Au is then deposited, as a standard process for board viability [15]. Although this improves biocompatibility, previous studies have indicated that exposed Cu-based structures typically show an increase in electrochemical noise due to corrosion, in relation to homogeneous Au surfaces [16,17]. The impact of electrochemical noise in standard PCB-based electrodes, in the context of large-area MEAs, has not been previously investigated.

This work demonstrates for the first time a transformation of a static, large-electrode-area MEA to a continuous flow, low-noise system. A custom-made microfluidic network was designed, enabling efficient nutrient delivery and waste removal across the whole electrode area. Prototype fabrication and fluidic functionality of the device are outlined, exploiting a PCB version of the previously demonstrated MEA aiming to leverage the associated low-cost production and integration. Section 2 shows the methodology behind the design and manufacturing process of the microfluidic MEA, as well as the proposed setup for conducting noise recordings. Section 3 highlights the implementation of the final prototype and initial electrical characterisation of the device. Section 4 and Section 5 contain a discussion of the results, including future work required.

## 2. Materials and Methods

Different designs for a microfluidic network compatible with the geometrical characteristics of a large-area MEA and electrical measurement setup were generated using commercial CAD software (AutoCAD^®^ 22.0). The software package COMSOL Multiphysics^®^ 5.3a was used to simulate the concentration profiles of the designed microfluidic structures under steady flow and select the one resulting in the largest uniformity. For all models, COMSOL stationary studies were used for creeping flow and transport of diluted species. The inlet flow rate, concentration, diffusion constant, and viscosity of the liquid were selected to represent experimental conditions for the flow of the growth medium for cells (100 μL/min, 1 mM, 1 × 10^−9^ m^2^/s, and 0.5 m^3^/mol^2^, respectively). An extra-coarse mesh was exploited with a “Free Quad” symmetry face, optimised for fluid dynamic studies (maximum element size 600, minimum element size 120, maximum element growth rate 1.3, curvature factor 0.9, and resolution of narrow regions 0.4).

Once the optimum microfluidic design was selected, the structure design was rapid prototyped and tested for fluidic tightness. A 0.05 mm thick PMMA sheet was sandwiched between two layers of double-sided adhesive film (3M 468MP). The final microfluidic design was laser-cut out onto the 0.5 mm PMMA and adhesive assembly. A lid, with the corresponding input and output hole, was laser-cut on 5 mm thick PMMA. The patterned PMMA and adhesive structure were stuck onto the 5 mm lid by removing one side of the double-sided adhesive film. This assembly was then adhered onto the MEA micro-chip by removing the second side of the film. The MEA device was designed in a standard PCB design CAD software (Altium Designer ^®^ 17.0), comprising a gold-plated sensing electrode layer. The sensing layer consisted of eight planar, circular electrodes of four different surface areas. The dimensions of the MEA were replicated from a previously exploited MEA on glass substrate, so that the same electrical interfacing apparatus could be exploited for low-noise brain cell signal recordings [10]. A PCB-based substrate was used in this instance instead of glass due to the cost effectiveness, potential for large-scale manufacturing, and ease for further electronics integration. The surface roughness as measured with a DEKTAK surface profilometer amounted to approximately 100 nm. Hence, the electrode area could be taken equal to the geometrical value of the circular electrodes. The areas used amounted to 1 mm^2^, 2 mm^2^, 7 mm^2^, and 12 mm^2^. The glass-reinforced epoxy laminate FR4 had a thickness of 800 µm, and it supported an Au plated layer above 35 µm of Cu. The functionality of the completed prototype was tested using a dye solution injected through the device inlet to verify its fluidic tightness, as well as uniform coverage of all electrodes. 

Electrical voltage noise recordings were carried out on the manufactured chips using static and continuously flowing KCl solution. The conductivity of 100 mM aqueous KCl was calculated in a previous study to be approximately 200 Ω·cm, which is in close agreement with Sigma Aldrich’s reference conductivity value for culture medium [10]. The solution resistance, modelled in detail previously, decreases when the molarity increases, which leads to a gradual change of the Maxwell–Wagner relaxation frequency for OOC systems. Hence, 100 mM KCl solution was also used in this work as an approximation to cell culture medium for the purpose of electrical noise measurements. Flow rate control was achieved with a microfluidics system, consisting of a pressure pump (OB1 MK3+; Elveflow, Paris, France [18]) and digital flow sensor (MFS3; Elveflow, Paris, France [19]) capable of measuring flow rates up to 80 µL/min. Similarly, prior microfluidics systems have used flow rates ranging from 1 µL to 1 mL/min [20,21,22]. The microfluidic MEA was placed in a container and connected to a voltage amplifier (Brookdeal Preamplifier 5006; Ortec, TN, USA), high-speed analogue-to-digital converter (ADC) (NI USB-6343; National Instruments, Austin, TX, USA), and a device for minimising mains (50 Hz) interference (Humbug; Quest Scientific, Vancouver, BC, Canada). The chip container, flow sensor, and voltage amplifier were contained within a Faraday cage to minimise interference in the recordings. Voltage recordings were conducted at a sampling rate of 125 kHz and an amplifier gain of 1075. The recordings were downsampled to 50 kHz for offline analysis and the power spectra were further filtered with a moving mean (window length of 5) for clarity.

## 3. Results

### 3.1. Concentration Profile

**Figure 1:**
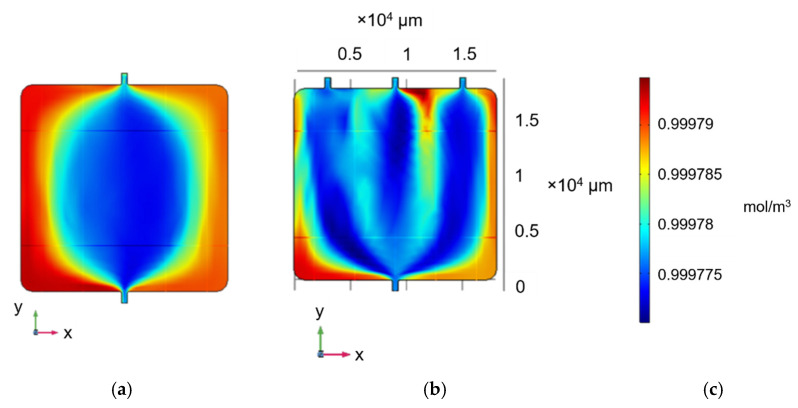
Concentration profile (mol/m^3^) of square continuous-flow chamber featuring (**a**) one inlet and one outlet, and (**b**) three inlets and one outlet at steady state. (**c**) Concentration range for (**a**,**b**).

The microfluidic MEA developed for the purposes of this study was designed to ensure both continuous and uniform flow of growth medium across the complete surface of the chip. Alternative geometries were designed via COMSOL, and the concentration profile was modelled considering an inlet flow rate of 100 μL/min. The initial design comprised a single inlet and outlet, positioned in the middle of the opposite sides of the chamber (Figure 1a). This configuration resulted in accumulation of liquid at the corners and edges of the MEA area. This nonuniformity in the cell culture medium flow over the MEA surface is expected to be particularly detrimental for future cell-based studies. In particular, this design could result in cell death on the corners and edges of the MEA due to the effects of cell metabolism, depletion of nutrients at the corners of the device, and excess waste accumulation. To mitigate this issue, two additional inlets were added symmetrically to the original one (Figure 1b).

**Figure 2:**
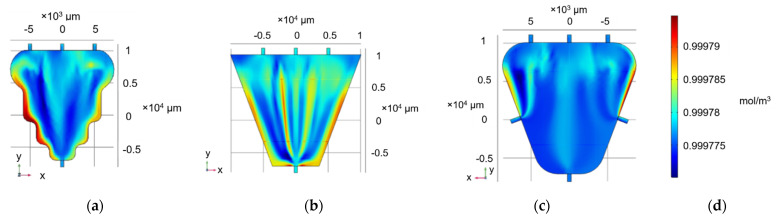
Concentration profile (mol/m^3^) of trapezium continuous-flow chamber featuring three inlets and one outlet (**a**) with a curved profile following electrode geometry and (**b**) straight edges (**c**) featuring three inlets and three outlets. (**d**) Concentration range for (**a**,**b**,**c**).

Although the accumulation of liquid at the top two corners was reduced, there were still low-flow spots between the inlets and at the bottom two corners. Considering the addition of more inlets impractical for implementation, the three-inlet approach was chosen, but the chamber shape was modified from square to a trapezium, following the MEA electrode pattern and theoretically minimising accumulation in the bottom corners. Two alternative versions were simulated, one following, in direct proportionality, the electrode contour (Figure 2a) and one with straight sides tangentially covering their complete surface (Figure 2b).

In Figure 2a,b, the accumulation of liquid in the bottom was minimised; nonetheless, there was slight accumulation in the edge of the trapezium side faces. For this reason, a final modification was simulated (Figure 2c). The three-inlet trapezium approach was maintained; however, rounding the edges with a 3 mm radius facilitated a smoother flow, and adding two symmetrical outlets in the middle of the side edges minimised liquid accumulation on the edges.

### 3.2. Microfluidic Prototype Implementation

The identified optimal microfluidic chamber geometry was implemented in the final device prototype. The final device should also be compatible with the low-noise laboratory measurement setup. To this end, (1) the height of the device could not exceed 30 mm, (2) there had to be an inlet and outlet hole within chip area, and (3) the inlet and outlet channels had to be of equal length and width, to assure synchronised liquid flow over the chip.

**Figure 3:**
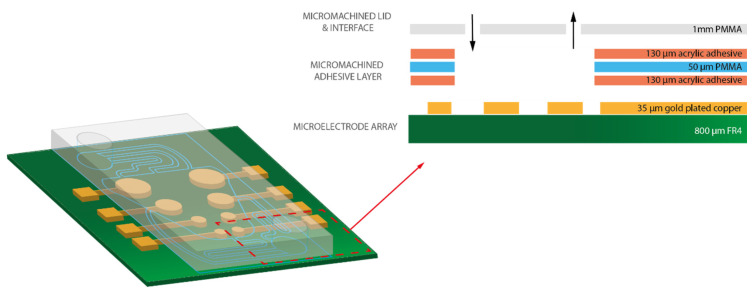
Three-dimensional model of the prototyped continuous flow microfluidic MEA platform. The sensing part of the device consists of four electrode pairs with the areas 12, 7, 2, and 1 mm^2^, and electrode spacing of 9, 7, 3, and 2 mm, respectively (measured from the centres of each electrode). The inset shows a magnified view of the highlighted cross-section, in dashed red lines, comprising the following material layers: FR4 (green), gold-plated contacts and electrodes (yellow), acrylic adhesive (orange), 50 µm PMMA (blue), and 1 mm PMMA for the lid (gray).

**Figure 4:**
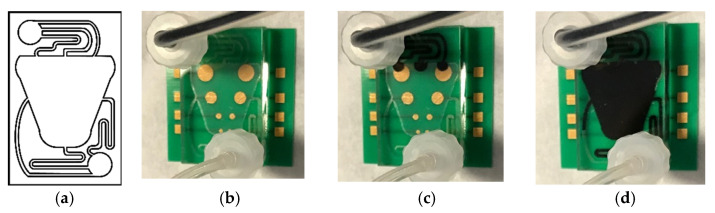
(**a**) Microfluidic network design. (**b**–**d**) Assembled microfluidic MEA tested for fluidic tightness under continuous flow using a dye (**b**) when the device chamber was empty, (**c**) when the device chamber filled through the inlet ports, and (**d**) when the dye moved through the centre and into the outlet ports.

A model of the chosen device geometry and materials are shown in Figure 3. For this device, the design depicted in Figure 4a was developed, housing two standard Luer-type microfluidic connectors for the efficient interfacing of the chip to the macroscopic world. This design was laser-micromachined in the PMMA double-sided tape sandwich described in Section 2. The micromachined pattern was then adhered on the PCB MEA substrate and finally sealed with the 5 mm PMMA lid, housing the inlets for the microfluidic connectors. The connectors were screwed in the inlets and tubings attached to them to deliver the dye solution. On some instances, there was accumulation of bubbles in several locations on the chip. When this was observed, the device was placed in an O_2_ plasma chamber (Zepto Model 2, Diener Electronic, Ebhausen, Germany) and treated for 15 s at 100 mW in order to become more hydrophilic and facilitate bubble-free initial wetting of the chip surface (Figure 4b–d).

### 3.3. Electrical Characterisation

**Figure 5:**
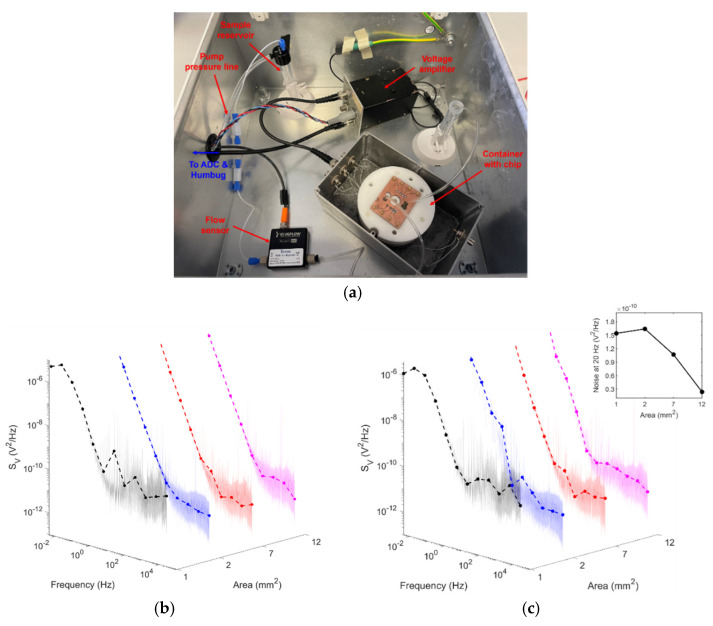
(**a**) Microfluidics setup within Faraday cage, which includes the sample reservoir, microfluidic flow sensor (MFS3; Elveflow), voltage amplifier (Brookdeal Preamplifier 5006; Ortec), and a custom-built container for the MEA and microfluidic tubing. The chip container was covered with aluminium foil for experiments and the Faraday cage was closed. (**b**,**c**) Power spectra of all electrode areas (**b**) under a static solution, and (**c**) at a continuous flow rate of 80 µL/min. Inset: Change in noise at 20 Hz across all electrode areas under continuous flow.

Voltage recordings were conducted on all electrode areas at zero flow (0 µL/min) and the maximum sensor flow rate (80 µL/min) using the setup shown in Figure 5a. No significant change in electrical voltage noise was seen when transitioning from no flow to flow (this change varied between 1.2 × 10^−10^ and 1.9 × 10^−9^ V^2^/Hz). Power spectra for each area at a flow rate of 80 µL/min are shown in Figure 5b. At thermodynamic equilibrium, at zero volt overpotential, the voltage power spectral density is given as follows [10]:

(1) $$S_{V}\left(\omega \right)=4\mathrm{kTZ}’\left(\omega \right)$$

where Z’ is the real component of the impedance, T is the temperature, k is the Boltzmann constant, and *ω* is the angular frequency. Given that the electrodes in question are not ideally polarizable, the double-layer capacitance was described using a constant phase element (CPE), and the impedance as a function of CPE was then used to model the expected noise of an electrode/electrolyte system. This was investigated for the case of a metal/electrolyte interface for Au/Electrolyte in prior work [10]. According to Equation (1), with increasing electrode area and, hence, decreasing impedance, voltage power spectral density should decrease. This is highlighted in the inset of Figure 5b with the quantified voltage power at 20 Hz. These results indicate that the Cu/Au electrodes showed a larger baseline noise level in comparison to Cr/Au electrodes on a glass substrate; however, despite this, the voltage noise was kept, at all times, below 30 µVpp (with amplifier RMS noise of 1.13 µVpp at a bandwidth of 125 kHz). Although mains interference was minimised using the Humbug and Faraday cage, it is possible that other interfering signals may have been present in the recordings from surrounding equipment, given that the Faraday cage could not be completely shut with microfluidic tubing and electrical wiring in place, as well as that the ADC and Humbug had to be placed outside the cage.

## 4. Discussion

In order to transition from static MEA structures to continuous-flow ones, the design of the microfluidic structure needs to be carefully selected, assuring a uniform flow across the complete chip surface; nonuniformities in flow could result in preferential cell growth in specific parts of the array [23], thus biasing the electrical recordings. The practical aspects need to also be carefully considered, enabling the exploitation of low-noise measurement setups for reliable cell signal recordings. It is also very critical to assure a bubble-free operation under flow since the presence of bubbles over the electrodes will compromise the signal transduction. In this work, a systematic study was described which efficiently achieves all of the above, exploiting a PCB-based MEA. Electrical characterisation tests were performed on the chip under both static and continuous flow, indicating that an increase in flow rate did not significantly influence the recoding background noise, where a plateau of 30 µVpp was maintained. Future work includes modelling nutrient consumption within the system to ascertain how parameters such as velocity, wall shear stress, and critical perfusion rate are affected and, hence, the suitability of the device to different cell types [24,25]. These simulations should be run in tandem with experimental cell work, given the inherent complexity associated with modelling cells. Lastly, biocompatibility studies should be performed using cellular cultures, and further electrical characterisation over a broader range of flow rates should be conducted. 

## 5. Conclusions

In this work, the transition from static MEAs to continuous-flow ones, towards more realistic Brain-on-Chip structures, was described. Different microfluidic designs were simulated, aiming for uniform growth medium distribution across a previously demonstrated, custom-made MEA for cell electrical sensing. The optimum design comprised a three-inlet, three-outlet rounded-corner microfluidic chamber following the electrode periphery. The design was integrated on a PCB-fabricated MEA for the first time, and a plateau below 30 µVpp was achieved when changing from a static solution to a maximum flow rate of 80 µL/min. The observed change in voltage noise was minimal when changing from a static KCl solution to a continuously flowing one. Future work will include electrical characterisation of the device under continuous flow in a wider range of flow rates along with signal recordings with electrogenic cells.
